# ‘*Every Touch Point Is an Opportunity*’: Tobacco Control Experts' Views on How to Implement Smoking Cessation Interventions Within an Australian Lung Cancer Screening Program

**DOI:** 10.1002/cam4.70963

**Published:** 2025-05-19

**Authors:** Nathan J. Harrison, Diane Riddiford‐Harland, Sarah York, Henry M. Marshall, Joel J. Rhee, Emily Stone, Mei Ling Yap, Ashleigh R. Sharman, Marianne Weber, Susan A. McCullough, Terri Byrne, Christine L. Paul, Jacqueline A. Bowden, Billie Bonevski, Nicole M. Rankin

**Affiliations:** ^1^ Flinders Health and Medical Research Institute, College of Medicine and Public Health Flinders University Adelaide South Australia Australia; ^2^ NHMRC Centre of Research Excellence on Achieving the Tobacco Endgame, School of Public Health The University of Queensland Herston Queensland Australia; ^3^ National Centre for Education and Training on Addiction (NCETA), Flinders Health and Medical Research Institute, College of Medicine and Public Health Flinders University Adelaide South Australia Australia; ^4^ Graduate School of Medicine, Faculty of Science, Medicine and Health University of Wollongong Wollongong New South Wales Australia; ^5^ Discipline of General Practice, School of Clinical Medicine, Faculty of Medicine and Health UNSW Sydney Kensington New South Wales Australia; ^6^ Centre for Health Policy, Melbourne School of Population and Global Health University of Melbourne Parkville Victoria Australia; ^7^ University of Queensland Thoracic Research Centre and Department of Thoracic Medicine The Prince Charles Hospital Chermside Queensland Australia; ^8^ Department of Thoracic Medicine and Lung Transplantation St. Vincent's Hospital Sydney Darlinghurst New South Wales Australia; ^9^ School of Clinical Medicine, Faculty of Medicine and Health UNSW Sydney Kensington New South Wales Australia; ^10^ Melbourne School of Population and Global Health University of Melbourne Melbourne Victoria Australia; ^11^ Collaboration for Cancer Outcomes, Research and Evaluation, Ingham Institute UNSW Sydney Liverpool New South Wales Australia; ^12^ George Institute for Global Health UNSW Sydney Barangaroo New South Wales Australia; ^13^ Liverpool and Macarthur Cancer Therapy Centres Western Sydney University Campbelltown New South Wales Australia; ^14^ Sydney School of Public Health, Faculty of Medicine and Health The University of Sydney Camperdown New South Wales Australia; ^15^ The Daffodil Centre, the University of Sydney, a Joint Venture With Cancer Council NSW, Faculty of Medicine and Health The University of Sydney Sydney New South Wales Australia; ^16^ Consumer Panel, Thoracic Oncology Group of Australasia Thornbury Victoria Australia; ^17^ Consumer Member, TROG Cancer Research Waratah New South Wales Australia; ^18^ PC4 Community Advisory Group, Primary Care Collaborative Cancer Clinical Trials Group Melbourne Victoria Australia; ^19^ School of Medicine and Public Health The University of Newcastle Callaghan New South Wales Australia; ^20^ Hunter Medical Research Institute New Lambton Heights New South Wales Australia

**Keywords:** Australia, early detection of cancer, lung neoplasms, smoking cessation, tobacco control

## Abstract

**Background:**

Targeted lung cancer screening (LCS) presents unique opportunities for smoking cessation among high‐risk individuals. Implementation requires consideration of context‐specific determinants. We sought perspectives from tobacco control/smoking cessation experts on optimally implementing smoking cessation interventions in an Australian LCS program.

**Methods:**

Recruitment was via maximum variation and snowball sampling in 2022. Individual/pair interviews explored factors that may influence acceptability and feasibility, potential delivery models, and implementation strategies. We interpreted interview data using codebook thematic analysis, then mapped key topics against the Consolidated Framework for Implementation Research and previous LCS expert‐identified determinants.

**Results:**

Expert participants' (*N* = 30) roles included program/policy managers, researchers and clinicians, working in academia, not‐for‐profits/peak bodies and health departments. They emphasised the benefits of integrating smoking cessation interventions into routine LCS encounters. Key barriers included perceived professional and LCS participant reluctance, stigma, and rural access. Experts discouraged duplicating current cessation resources, and suggested prioritising implementation efforts. Flexible delivery models, addressing needs of priority populations (e.g., rural/remote, culturally and linguistically diverse), were recommended. Experts generally favoured a ‘hybrid’ intervention pathway, combining internal and external cessation services, to maximise feasibility and uptake. Training program staff on cessation intervention delivery and leadership buy‐in were emphasised as essential requirements.

**Discussion:**

In this Australian tobacco control expert sample, there was near‐unanimous support for embedding cessation interventions within routine LCS delivery and capitalising on opportunities to overcome current service delivery barriers. In conjunction with LCS participant perspectives, findings are relevant to planning and implementing cessation interventions in LCS contexts.

## 1 Introduction

Tobacco control has averted substantial lung cancer mortality in Australia [1]. Lung cancer, however, remains the leading cause of cancer mortality with considerable burden projected in coming decades [2]. Among higher‐risk participants with tobacco smoking history, randomised trials of lung cancer screening (LCS) via low‐dose computed tomography (LDCT) have reduced lung cancer‐specific and all‐cause mortality [3, 4, 5]. LCS participation was also associated with increased readiness to quit [6] and smoking cessation [7], and evidence generally refuted concerns that receiving LCS results could provide ‘license’ or *encourage* smoking [8, 9]. Providing smoking cessation interventions in LCS has been recognised as essential to maximise the benefit and cost‐effectiveness of screening [10]. Australian pre‐implementation research has noted unique intervention opportunities throughout the LCS screening and assessment pathway, such as when promoting LCS or discussing smoking history in shared decision‐making [11, 12, 13, 14].

Ongoing international research considers how to routinely deliver and integrate smoking cessation interventions in LCS, for example [15, 16]. Along with effectiveness and cost‐effectiveness, acceptability and accessibility have been identified as key determinants, including how to minimise complex barriers such as smoking‐associated stigma and ensure equitable reach [17, 18, 19]. Optimised integration in new LCS programs will require consideration of trial effectiveness‐implementation data (e.g., on intervention timing and delivery) [20, 21], and formative research to understand potentially unique determinants within the local context.

Australia's Government‐funded National LCS Program commences in 2025, for asymptomatic individuals meeting age (50–70 years) and smoking history (> 30 pack‐years, and < 10 years since smoking cessation) eligibility criteria [22]. Two‐yearly screening and interval LDCT scans will be mandatorily ‘bulk‐billed’ (without individual payment) by radiology providers under public insurance [23]. LCS participants may still incur variable out‐of‐pocket costs, including for travel and primary care appointments [24], as medical practitioners will assess eligibility and provide initial referral. Repeated health professional contact in the Program will also facilitate opportunities for smoking cessation‐related discussion. Current smoking prevalence among the LCS‐eligible population (estimated > 70%) [25] far exceeds the general population, reflecting the targeted criteria. Health service engagement is also typically lower [26, 27], highlighting the urgent need for cessation support.

LCS delivery models are varied across trials and international practice, including in the extent of cessation intervention integration. Whereas counselling and follow‐up support has been coordinated and delivered within the LCS program in a highly integrated approach, for example [15, 28], others have involved only brief intervention and referral [7]. ‘Hybrid’ models might also incorporate elements of both ‘embedded’ and ‘external’ services, where some intervention delivery (e.g., pharmacotherapy management) is embedded and external agency referral is also required [29]. In Australia, evidence‐based clinical guidelines recommend combination pharmacotherapy and behavioural counselling for best‐practice cessation support and recognise various intervention delivery options (outside LCS) [30, 31]. Although further implementation details are pending for the forthcoming National LCS Program, ‘the appropriate [smoking cessation] pathway’ has been described as providing existing local service information during eligibility assessments [32], and using existing Government‐funded services [33].

In earlier focus groups, experts with LCS‐related clinical, policy, cancer screening program, and research roles—but not necessarily LCS and/or smoking cessation professional familiarity in advance of the National LCS Program's announcement—strongly supported offering smoking cessation interventions in LCS [13]. We sought to expand on this understanding by recruiting an independent sample with tobacco control expertise from other (i.e., non‐LCS) contexts, and through a focus on smoking cessation intervention delivery and implementation considerations for Australian LCS.

### 1.1 Aims

We aimed to explore tobacco control experts' views on the role of smoking cessation support in LCS and how smoking cessation interventions could be optimally implemented in an organised Australian LCS program. Specific objectives were to explore views regarding:

1. Factors that may influence acceptability and feasibility, including implementation barriers and facilitators.
2. Potential delivery models and implementation strategies.

## 2 Methods

### 2.1 Design, Sample, and Recruitment

We conducted interviews in March–June 2022, that is, before the National LCS Program was announced, and described a ‘potential LCS program’. Participants were tobacco control and/or smoking cessation experts, including health practitioners, researchers, and/or policymakers. We purposively sought representation from varied professional backgrounds. An initial list of potential invitees was developed, through examining peer‐reviewed publications, University websites and publicly accessible documents, and from the multidisciplinary research team's knowledge of relevant organisations and individuals. Invitees were emailed a project outline with one, 2‐week follow‐up email. This was supplemented with professional social media and website posts, and during interviews participants were invited to recommend other eligible contacts for potential invitation. All participants were offered an AUD$50 retail voucher. We initially sought to recruit up to 40 experts, but considered that diverse professional perspectives were represented with 30 experts and ceased new recruitment. Sufficient information power was likely obtained with focused participant inclusion and experienced research academic interviewers [34].

### 2.2 Procedures

Procedures had ethical approval (including at the University of Sydney, 2021/850) and were first tested in two pilot interviews in December 2021–January 2022. Participants self‐reported characteristics through a brief online survey, with at least two email reminders if required. Participants were offered interviews or focus groups based on preference, either in‐person where feasible or via telephone/videoconferencing. Interviews were conducted by one of two female, PhD‐qualified senior research academics (D.R‐H., N.M.R.), with a mean duration of 47 min (range 17–62 min).

The full discussion guide appears in Additional File S1 (overview in Table 1), and was designed and reviewed based on the research team's clinical and research perspectives. Consistent with the potential Australian program parameters proposed at the time, eligible LCS participants were described as 55–74 years (or 50–74 years for Aboriginal and Torres Strait Islander people), with > 30 pack‐year smoking history and < 15 years smoking cessation [33].

**TABLE 1:** Overview of discussion guide.

Introduction	The introduction emphasised the targeted age‐ and tobacco smoking history‐based invitation and outcomes of landmark international LCS trials [3, 4], and that the ways to optimally embed smoking cessation intervention within LCS programs are unknown [14, 26]
	Organised LCS programs and pilot programs implemented in other countries (e.g., the United States and South Korea) were also described
Experts' experience working in tobacco control and smoking cessation	Nature of professional role(s) Years' experience Employment setting Views on effective components in *existing* smoking cessation programs (i.e., in contexts outside of LCS)
Perspectives on embedded smoking cessation interventions within LCS	Potential LCS participant preferences and responses to offers of intervention (e.g., refusal, opt‐out mechanisms), with reference as required to promising examples of cessation intervention from published reviews [35, 36] Specific considerations for LCS participants who either do not wish to quit or are from priority population backgrounds, and; implications for potential resource co‐design
Perspectives on potential approaches for smoking cessation programs	Expert views were elicited for three potential pathways (represented in Table 4). For each approach, prompts were used to elicit perspectives on likely success, and potential implementation facilitators and barriers
	Experts were also asked about the frequency of cessation advice they considered appropriate during initial and repeat LDCT scans, as well as alternate approaches, including the professionals that should be involved in providing cessation intervention and any required staff training
Perspectives on broader policy and health system considerations	Most important policy factors, and the level of scientific evidence required (e.g., international trials and follow‐up data, appropriate outcomes, cost‐effectiveness considerations), to support optimally embedded cessation intervention in LCS Most significant barriers and enablers to successful implementation, and most significant challenges for service delivery (e.g., transfer to different settings including follow‐up after mobile LCS) Pathways to increase LCS awareness among, and offer LCS to, potentially eligible consumers in existing smoking cessation programs
Other data items^a^	The potential use of e‐cigarettes/vaping as smoking cessation interventions in an LCS program

Abbreviations: LCS, lung cancer screening; LDCT, low‐dose computed tomography.

^a^ These data are not included in the current analyses; as listed, they were typically asked at the end of interviews and were assumed to have little influence on experts' other (tobacco smoking cessation‐related) views in interviews.

### 2.3 Analysis

Verbatim transcripts were produced using Trint, verified against audio by N.J.H. for familiarity, and edited as necessary. Participants were not offered transcripts for review, but ‘off‐the‐record’ statements were removed if requested during interviews. Identifying and contextual information (e.g., location or health service) was anonymised.

We interpreted interview data using a codebook thematic approach [37, 38]. First, six researchers (N.M.R., D.R‐H., S.Y., A.R.S.) and consumer advocates (S.M., T.B.) individually and inductively coded at least one of two exemplar transcripts line by line, following Coates et al. [39] N.J.H. subsequently coded these same two transcripts and incorporated observations from across the analyst team in a preliminary codebook. The codebook was iteratively refined through individual coding by two authors on each of a further three transcripts (N.J.H., S.Y., S.M.). The final codebook, shown in Additional File S1, was applied using NVivo 14 (Lumivero, 2023). Similar code patterns were identified as candidate themes [39] and described in detailed topic summaries.

To interpret factors that may influence acceptability and feasibility (objective 1), we narratively synthesised key themes and topics. We then mapped these against key findings from our previous study with LCS‐related experts [13], and a widely used implementation determinant framework, the updated Consolidated Framework for Implementation Research (CFIR) [40]. Potential delivery models and implementation strategies were summarised (per objective 2) following their hierarchical codebook grouping, and mapped against key timepoints of the proposed LCS screening and assessment pathway [41] or as likely ‘cross‐cutting’.

## 3 Results

### 3.1 Expert Characteristics

*N* = 30 experts participated in 28 interviews. There were two separate interviews with colleague pairs. Available professional/personal characteristics (from *N =* 23 complete surveys; Table 2) indicate the sample was highly experienced: 63% reported > 20 years' professional experience. Most experts worked in program/policy manager or research roles (total 69%), and academic or not‐for‐profit/peak body settings (total 53%). Experts reported residence in each Australian state/territory, plus Aotearoa New Zealand for two experts identified in snowball sampling as having additional relevant expertise (e.g., in binational organisations, senior health service roles).

**TABLE 2:** Professional and personal characteristics of expert interviewees.

Professional characteristics^a^	*n* (%)
Time spent professionally employed, in years (*N* = 27)	
6–10	2 (7%)
11–15	3 (11%)
16–20	5 (19%)
21–25	5 (19%)
26–30	6 (22%)
> 30	6 (22%)
Professional role (*N* = 29)	
Program manager/policy manager	10 (34%)
Researcher	10 (34%)
Nurse/other clinician (e.g., medical practitioner, pharmacist)	6 (21%)
Smoking cessation counsellor	1 (3%)
CEO/director	1 (3%)
Other (advisor)	1 (3%)
Workplace setting (*N* = 30)	
Academic/university‐based	8 (27%)
Not‐for‐profit organisation/peak body organisation^b^	8 (27%)
Government health department	5 (17%)
Hospital	4 (13%)
Clinic	2 (7%)
Combination of settings above	3 (10%)
Location of residence (*N* = 30)	
Australia	28 (93%)
New South Wales	13 (43%)
Victoria	4 (13%)
Tasmania	3 (10%)
Queensland	3 (10%)
South Australia	2 (7%)
Australian Capital Territory	1 (3%)
Northern Territory	1 (3%)
Western Australia	1 (3%)
Aotearoa New Zealand	2 (7%)
Personal characteristics^c^	
Age in years (*N* = 23)	
18–30	1 (4%)
31–40	5 (22%)
41–50	7 (30%)
51–60	8 (35%)
61–70	2 (9%)
Gender (*N* = 30)	
Female	22 (73%)
Male	7 (23%)
Other gender identity	1 (3%)
Country of birth (*N* = 23)	
Australia	14 (61%)
Other country	9 (39%)
Aboriginal or Torres Strait Islander person (*N* = 23)	
No	22 (96%)
Yes	1 (4%)

^a^ After one emailed survey reminder, available professional characteristics were extracted from interview transcripts or publicly available websites, and participant verification was sought.

^b^ Reported using response option ‘peak body’, typically indicating in Australia a non‐governmental organisation that advocates (e.g., on health issues) on behalf of individual and organisational members. Reporting of nominal outcome categories is by descending frequency. Potential categories of professional characteristics are collapsed where possible to avoid small cell sizes (approximately < 10% of sample).

^c^ Only self‐reported personal characteristics are presented here.

### 3.2 Objective 1: Factors That May Influence Acceptability and Feasibility, Including Implementation Barriers and Facilitators

Table 3 includes an overview of key themes (corresponding to subheadings below) and topics (in bold type), with illustrative quotations and aligned CFIR constructs. Points of convergence/divergence with our earlier LCS‐related expert study [13] are also noted.

**TABLE 3:** Summary of key themes and topics related to factors that may influence acceptability and feasibility from analysis of experts' perspectives, including implementation barriers and facilitators, with corresponding CFIR constructs and alignment with previous LCS‐related expert views.

Key themes and topics from coding	Illustrative quotations	Aligned CFIR domain (subdomain): CFIR construct [40]	Convergence/divergence with LCS‐related experts' views [13]
Evidence for smoking cessation, within and outside of LCS			
Tobacco control environment (±)	“We were actually *pretty* good at tobacco control…We *know* how to help people quit smoking. We *know* how to drive down smoking rates. We just have to choose whether we're going to do it or not.” *[#06, researcher]*	Outer setting: Local conditions	n/s
Evidence base (+)	“It's talking about things that work.” *[#03, program manager]*	Innovation: Innovation evidence‐base	n/s
Implementation challenges (−)	“…there are stacks of resources around smoking cessation and adding to the environment of many, many resources is probably not valuable.” *[#04, program manager]*	Implementation process: Adapting	LCS experts also suggested adapting resources to the Australian context
Learning lessons from implementation experience (+)	“I think it's obviously much easier to make those changes initially, than set something off and then make those modifications… We make the same mistakes that other districts—other countries—have made when we could have accessed that information initially….” *[#26, clinician]*	Innovation: Adaptability	LCS experts also noted existing models (from LCS/non‐LCS contexts) as potentially suitable, with appropriate adaptation
LCS participation considerations from experts' perspectives			
Attitudes among LCS participants (−)	“People don't need just more information and education, I think most people actually know that smoking is not good for them.” *[#13, researcher]*	Individuals (Roles): Innovation recipients	LCS experts also suggested approaches sensitive to LCS participants' beliefs, but with greater emphasis on discussion timing and stage of change
	“Whatever that *helps them* to see, ‘ah—I can make changes’.” *[#19, program manager]*		
Motivation among LCS participants (±)	“I think you're dealing with a population that's already self‐selected… You're more motivated than just someone who sees a warning on their cigarette pack every day and goes, ‘urgh, I don't like the look of that’…” *[#06, researcher]*	Individuals (Roles): Innovation recipients	LCS experts suggested LCS participants could be more health‐conscious (than eligible individuals who do *not* participate in LCS), but with less emphasis on potential differences by current smoking status
Existing healthcare system engagement (+)	“With the population…it's catching them in at places that they are engaged with. So, this is a great example of if they're attending for something else… They already have [a] relationship…” *[#11, researcher]*	Outer setting: Partnerships and connections	LCS experts also suggested that existing (cessation) services could facilitate LCS participation, but with less emphasis on formal recruitment strategies
Access (−)	“But it's essentially: the more things that they have to do to get treatment, the less likely it is that they're going to quit.” *[#07, researcher]*	Implementation process: Assessing needs [Innovation recipients]; Inner setting: Structural characteristics [Physical infrastructure]	LCS experts also emphasised key access barriers for LCS participants, particularly cost
LCS is aligned with smoking cessation (+)	“I think it's acceptable. I think it's feasible. I think it's the correct and right thing to do… I think it needs to be…a fundamental part of the core values and mission and directive… It's not an add‐in. It's not optional.” *[#11, researcher]*	Inner setting: Mission alignment	LCS experts suggested that other professionals would expect alignment (but without specific reference to LCS participants)
Priority population and community considerations	“…it needs to be co‐designed with communities so that it is actually culturally appropriate, in terms of health literacy, in terms of language… We've got to make sure that any program that was initiated didn't make inequities worse.” *[#16, CEO/director]*	Implementation process: Assessing needs [Innovation recipients]; Implementation process: Tailoring strategies	LCS experts suggested tailoring cessation supports to increase priority population engagement, but with less emphasis on broader determinants and less recognition of co‐designed approaches
	“…it's about being able to refer people to somewhere that's going to be appropriate for them…it's about knowing what's right for various priority populations.” *[#27, researcher]*		
Health professional considerations from experts' perspectives			
Professional attitudes (−)	“I'm not sure too if people are really onboard working in that area already…they're like, ‘yeah, absolutely we'll screen for lung cancer all day, every day’, but then feel a little bit uncertain about offering smoking cessation support…” *[#01, program manager]*	Individuals (characteristics): Capability	LCS experts also suggested that some professionals would be personally reluctant to offer cessation support
Professional motivation (+)	“…in one of our main hospitals here there's a very keen oncologist, and a very keen anaesthetist, and a surgeon, who have started up a bit of a smoking cessation clinic within the hospital… and that was starting to get more oncologists understanding the importance of smoking cessation as part of cancer treatment.” *[#20, program manager]*	Individuals (characteristics): Motivation	n/s
Expectation of offering smoking cessation support at all LCS encounters (+)	“…everybody in services needs to take responsibility for this. So whether it's the radiographer, or the consultant radiologist who debriefs, or the nurse, it just needs to be a given that you cannot say, ‘this is not my problem’…” *[#24, clinician]*	Inner setting: Culture [Human equality‐centredness]; Inner setting: Culture [Recipient‐centredness]; Outer setting: Policies and laws	Some LCS experts also suggested that cessation supports should be routinely offered in LCS, but there was less emphasis on routine approaches in other (non‐LCS) contexts. LCS experts often described provision of interventions or active service as optimum care, but there was little recognition of clinical guidelines
Stigma			
Multi‐level impacts (−)	“I've always found it really horrible how stigmatised lung cancer is, and the victim blaming and ‘oh, you brought this on yourself. You should have known better. You should have quit’.” *[#06, researcher]*	Outer setting: Local attitudes	LCS experts also perceived high community and individual levels of stigma (strong convergence between studies), but less emphasis on specific enablers of stigma‐reducing communication
	“But if it's not negative, if it's just taken in a matter of fact, ‘right…’, without any moralising or judgement around it, then it puts it into the problem‐solving category rather than a weakness or a bad behaviour category.” *[#12, advisor]*		
Countering nihilism and fatalism (−)	“A really clear message that there is still a benefit from quitting at that stage and that it is actually possible—or potentially possible—for them to do that, particularly with evidence‐based supports increasing their chances of quitting.” *[#02, researcher]*	Individuals (Characteristics): Need	LCS experts also described potential LCS participant experience of nihilism/fatalism, but not specific strategies to reduce the impact
Systems considerations			
System‐level barriers (−)	“…if it's a busy environment…or competing priorities, then it just will fall to the bottom of the pile.” *[#01, program manager]*	Inner setting: Available resources	LCS experts placed particular emphasis on time constraints, including in specific work settings; strategies to enhance efficiency were not discussed
Cost (−)	“It's probably the view that smoking cessation has such low quit rates. You know, ‘are you really going to get return on investment…?’ Smoking cessation is one of the most cost‐effective interventions that we have… [but] someone might challenge you.” *[#10, researcher]*	Inner setting: Available resources [Funding]	LCS experts also perceived generally modest costs for cessation supports, and recommended no‐cost pharmacotherapy providing (strong convergence between studies)
Policy (+)	“Things are supported in different states, so being able to have something that's consistent…making sure there is a mechanism so that [it] is identified as coming…from the lung cancer screening program, and this is the pathway and the offerings…” *[#14, policy manager]*	Outer setting: Policies and laws	n/s
Smoking cessation in an LCS context can reinforce broader responses (+)	“I think it would be useful to reinforce any messages around cessation at the screening appointment, or however that works.” *[#17, program manager]*	Outer setting: External pressure [Societal pressure]	LCS experts also suggested that continuing population‐level responses was important, but with less emphasis on potentially supporting individual cessation efforts

*Note:* Where determinants were considered primarily by experts as barriers (−) and/or facilitators (+), the key topics are denoted as such. ‘n/s’ indicates that this topic was not substantively discussed by LCS‐related experts [13]. Illustrative quotations are labelled with sequential interview participant numbers and participants' broad professional roles. Smart verbatim quotations are edited for clarity and conciseness, with ‘…’ indicating removed text and text in [square brackets] inserted for context.

Abbreviation: LCS, lung cancer screening.

#### 3.2.1 Evidence for Smoking Cessation, Within, and Outside of LCS

Australia's existing *tobacco control environment*, including policy implementation and comparatively low smoking prevalence, was described as a facilitator of smoking cessation efforts. Remaining challenges were suboptimal cessation rates among those with established smoking, and misperceptions that tobacco control had been achieved (‘*done and dusted*’ [#18, researcher]) with less attention now required.

Experts described the current *evidence base* for LCS and smoking cessation, and felt that LCS implementation would be highly relevant and beneficial. In LCS, experts suggested consistently offering best‐practice cessation interventions, drawing on existing evidence from LCS/lung cancer care contexts where possible. Generating Australian LCS‐specific evidence was seen as a priority: experts assumed this could facilitate ‘*fairly subtle kind of changes to the standard…cessation support*’ [#02, researcher].

Significant *implementation challenges* were anticipated, and experts suggested adapting ‘generic’ resources and processes by considering frontline practitioners'/LCS participants' intervention delivery preferences. To avoid unnecessary re‐development, experts strongly supported *learning lessons from implementation experience* in overseas LCS. Services with sustained long‐term operations were highly regarded, but some practical barriers were noted for local adaptation.

#### 3.2.2 LCS Participation Considerations From Experts' Perspectives

A range of expected *attitudes among LCS participants* were primarily considered barriers. Experts assumed that targeted LCS could be stressful, causing some participants to feel ‘singled out’. To shape expectations, experts suggested featuring smoking cessation information in broader LCS communications but emphasising that LCS participation did not mandate quitting smoking. Many experts felt that historical acceptance of smoking should be recognised when working with current LCS‐eligible participants. They felt that participants would have likely attempted to quit previously, and therefore suggested positively affirming all cessation attempts, including if unassisted. Providing gain‐framed information on cancer risk reduction and correcting potential misperceptions (e.g., on self‐exempting beliefs) were considered valuable.

Many experts assumed that *motivation among LCS participants* to engage with initial and repeat screening would be lower for those with current (vs. former) smoking. However, experts anticipated that some of those engaged with LCS would likely be ‘ready to quit’. As a result, repeated contact was suggested to provide cessation intervention opportunities in line with participants' changing motivation, and in case of different responses to different professionals.

While noting that participants' *existing healthcare system engagement* would vary, experts suggested leveraging existing connections for LCS recruitment and smoking cessation. Experts held mixed views on the potential impact of providing cessation interventions on continued service engagement. To minimise disengagement, some suggested offering interventions as ‘*a leave‐piece…not a hard sell*’ [#14, policy manager] to those less receptive.

This sample described a range of participant *access* barriers, particularly in rural areas. Some experts were concerned about irregular mobile van LCS visits and recommended ‘*a portable team…alongside community support*’ [#23, clinician] for intervention delivery. Physical infrastructure was considered important in all areas, with ‘health hubs’ suggested to promote awareness.

Experts also anticipated LCS participants expecting *LCS is aligned with smoking cessation*, and therefore that intervention offers would largely be viewed positively (e.g., as ‘caring’). Although it was acknowledged that some LCS participants might resist smoking cessation‐related discussions, the expert sample largely felt that this concern could be overstated by clinicians in current practice, and categorised it as a ‘*non‐issue*’ [#24, clinician].

*Priority population and community considerations* were emphasised throughout interviews. Access and cost barriers were felt to have a disproportionate impact, especially for individuals with lower income. Experts also noted that for some culturally and linguistically diverse groups, there were higher perceived rates of pipe/hookah (especially in Arabic‐speaking communities), e‐cigarette, and cannabis use. As a result, they expressed concern about cigarette smoking alone defining LCS eligibility. The need for specific cessation interventions was discussed in relation to multiple priority populations, but especially in groups where smoking prevalence was highest, including for those experiencing homelessness, or mental health or substance use issues. Experts expected that many such LCS‐eligible individuals would have longer smoking histories, requiring more intensive interventions through multi‐session, face‐to‐face behavioural counselling and combination nicotine replacement therapies. Consideration was suggested of individuals' varied literacy and language abilities, experiences of intersectional disadvantage, and other health issues. To build trust and ensure cultural appropriateness, experts advocated for genuine pre‐implementation engagement and generally endorsed co‐designed, strengths‐based approaches, inclusive of family/community members. Existing targeted resources were described favourably, and adaptation and distribution through community networks was suggested.

#### 3.2.3 Health Professional Considerations From Experts' Perspectives

Experts expected smoking cessation intervention delivery to be generally acceptable and feasible across professional disciplines. However, experts identified *professional attitudes* as key barriers, including reluctance to offer cessation interventions when assumed to compromise patient relationships. They endorsed building confidence and promoting self‐efficacy (i.e., professionals' ability to provide benefit via cessation intervention delivery). The expert sample assumed those working in LCS would have particularly strong *professional motivation* and belief that LCS/smoking cessation would improve current lung cancer outcomes. For professionals in adjacent services, experts suggested incorporating LCS program‐specific considerations into evidence‐based communications and training.

Experts strongly endorsed an *expectation of offering smoking cessation support at all LCS encounters*, with clinical guidelines identified as facilitating best‐practice implementation. Citing cost‐effectiveness, health benefits, and a professional responsibility, experts strongly desired that routine intervention be an ‘obvious’ LCS component. Experts suggested that delivery by all clinical and trained non‐clinical staff would overcome perceived barriers and reinforce smoking cessation as ‘everyone's job’.

#### 3.2.4 Stigma

Strong community lung cancer stigma was perceived by expert interviewees, and it was noted that this partly reflected existing tobacco control strategies including anti‐smoking media. Experts considered addressing the *multi‐level impacts* of stigma to be important: they suggested that LCS communications avoid overt cancer/smoking cessation focus to avoid reinforcing stigmatising attitudes, and endorsed respectful and solution‐focused approaches. Experts recommended providing preferred wording that should and should not be used, including to contextualise cessation‐related discussion (e.g., ‘*why it's important for…your best care that this question is being asked*’ [#05, program manager]). To *counter nihilism and fatalism*, experts suggested reinforcing early cancer detection and smoking cessation benefits regardless of individuals' stage. Quitting being ‘too hard’ or ‘too late’ was often anticipated as LCS participant/professional perceptual barriers.

#### 3.2.5 Systems Considerations

Competing clinical priorities and time pressures were seen as existing *system‐level barriers* to providing cessation interventions. To overcome these, experts suggested drawing on defined roles with greater perceived capacity and explicit remit for cessation intervention delivery. Existing *cost‐*related barriers included limited funding for cessation‐focused roles and Medicare Benefits Schedule items. Cessation interventions were seen as highly cost‐effective, albeit sometimes resource‐intensive. Experts emphasised dedicated and consistent funding to support cessation interventions as an LCS requirement. Although sometimes contrasted with smoking's continued individual expenses, experts also recognised the time and opportunity costs required for LCS participants to engage with smoking cessation interventions. Experts strongly recommended providing pharmacotherapies with no/low participant costs to incentivise use.

*Policy* was consistently identified as an established facilitator of routine intervention integration—for example, some described the financial/accreditation implications of mandating smoking status documentation in primary care. Experts suggested leveraging broader cessation‐relevant policy to guide LCS efforts and promote policy leader buy‐in, but noted this should be combined with community engagement ‘on the ground’.

To support individual‐level intervention, experts also felt that *smoking cessation in an LCS context can reinforce broader responses* in tobacco control. They anticipated that population‐level strategies to reduce smoking prevalence could also ‘prompt’ LCS participants with current smoking but noted meaningful priority population inclusion and messaging consistency as important considerations.

### 3.3 Objective 2: Potential Delivery Models and Implementation Strategies

#### 3.3.1 Pathway Preferences

The three smoking cessation pathways presented to experts are represented in Table 4, with response summaries and illustrative quotations. Most preferred a ‘hybrid’ model, but experts repeatedly stated that all delivery models could have advantages and disadvantages.

**TABLE 4:** Overview of the three potential smoking cessation pathways discussed during expert interviews, and summary of responses.

Representation of potential pathway^a^ in the context of an organised LCS program	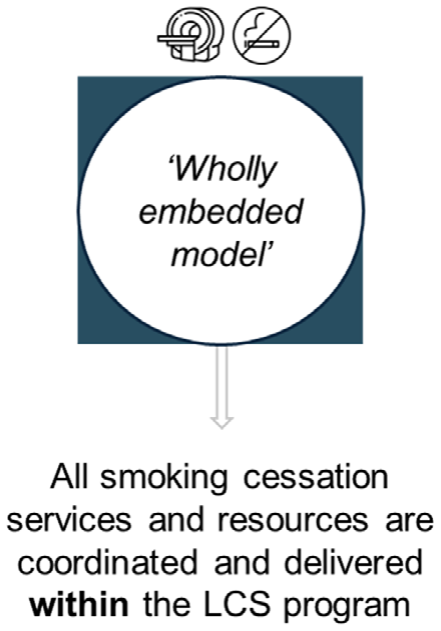	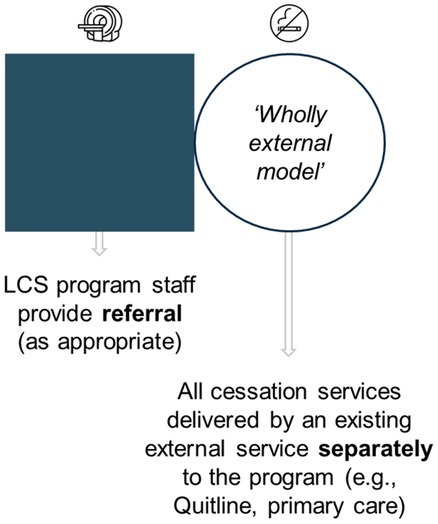	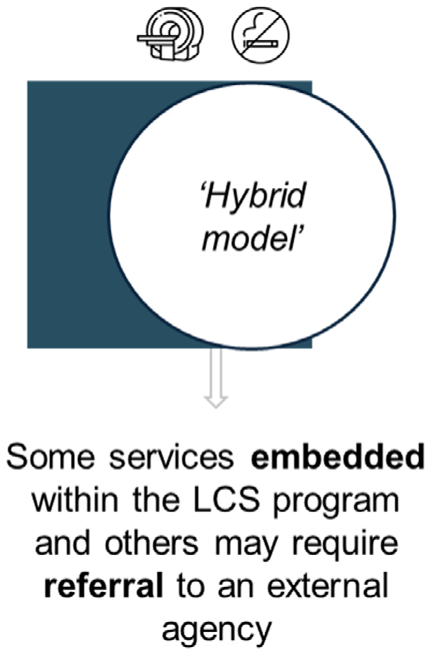
Summary of expert responses	Mixed support. Some experts endorsed the potential to provide dedicated services in this pathway, but others assumed resource‐intensiveness and therefore lower feasibility	No outright support. Experts considered that relying on external service delivery would result in lower engagement and uptake, compared to the other pathways	Supported by most. Experts perceived this to maximise existing service utilisation, cost‐effectiveness, and the extent of available interventions for delivery, plus minimise ‘back and forth’ referral
Illustrative quotations	“I feel like everything should be done in one place if possible. So we aren't asking them [LCS participants] to come here and go there… I would say that if they are coming for one appointment, might as well give them everything around it.” *[#28, nurse]*	“…you couldn't have the screening program and have the cessation message outsourced because… it separates it in the patient's mind. Because you're going into screening, ‘we care enough to screen you’, but not enough to help you quit—or ‘we'll help you, but we want someone else to do it’… Whereas if the message is clear that we really want to screen you and to show you that we'll get you started on the smoking cessation journey…” *[#12, advisor]*	“…the more support you can connect them with, the better, the more likely they are [to engage].” *[#04, program manager]*
	“I think doing it all in‐house might be challenging in that—how long is a person going to be in the program for all of those types of things?” *[#05, program manager]*		“…it needs to feel embedded to the individual, but it doesn't necessarily have to be. So, you might even call it the screening cessation thing, but it's done by the Quitline…” *[#25, researcher]*

^a^ The pathways vary in level of integration and are similar to those trialled in other LCS settings (e.g., Cinciripini et al. [29] including ‘integrated care’ and ‘quitline plus’ conditions). This figure has been designed using resources from Flaticon.com.

#### 3.3.2 Considerations by Pathway Stage

Table 5 includes illustrative quotations for key smoking cessation delivery models and implementation strategy considerations. There was general recognition that the broader LCS pathway's implementation would determine optimum cessation intervention delivery, with LCS engagement barriers at a given stage also influencing cessation intervention delivery. One expert noted *‘a lot of complexity, marrying up the recruitment…with smoking cessation treatment at the same time…’*, which required *‘two huge, different, bits of work’* [#12, advisor].

**TABLE 5:** Summary of expert‐identified considerations for smoking cessation delivery models and implementation strategies, mapped against key timepoints of the LCS screening and assessment pathway [41] with illustrative quotations.

Pathway stage				Cross‐cutting considerations
Identification and approach for referral	Risk assessment	Scan and receipt of results	Continuing in LCS program (and ‘beyond LCS’)	
“The main thing is to have people attend…having an environment that means people firstly see a reason to attend, but then are comfortable in continuing to attend.” *[#02, researcher]* “…every touch point in a lung cancer screening program is an opportunity for smoking cessation…” *[#16, CEO/director]*	“Integrating smoking care into usual care, as such a simple thing as on intake and assessment forms that when people get asked ‘How's your housing? How's your health? How's everything? Do you smoke? Yes, no?’, recording that status. And that becomes part of every conversation with people….” *[#19, program manager]*	“…there's probably interesting issues about when you do the smoking cessation advice, so that actually you run the screening first. And then, on the basis of the anxiety generated around the screening, you look to see where people are at…” *[#08, researcher]* “We need to maybe make them understand…that giving up smoking might lead to restoring lung health.” *[#28, nurse]*	“Generally speaking, we find that once people have met us and started to engage with us, they want to continue with us. We will do that as much as we can, but we obviously aren't resourced to follow up every individual 20 times….” *[#29, program manager]* “Just because the person might have been in—3 months ago, 6 months ago, or last week—doesn't mean that we shouldn't be asking them about smoking cessation [again]…” *[#18, researcher]*	Smoking cessation intervention types Service delivery modes Communication and messaging Other program requirements for smoking cessation, and other delivery considerations Workforce and training requirements

Abbreviation: LCS, lung cancer screening.

##### 3.3.2.1 Identification and Approach for Referral

Experts described LCS engagement and attendance as priority considerations–that is, to ensure that eligible participants would benefit from LCS and subsequent cessation intervention. They endorsed multi‐channel LCS communications to broaden reach and prompt potential participants' consideration of smoking cessation. Experts also supported communicating via smoking cessation programs. They considered that clients would be highly receptive, and that cessation counsellors could facilitate formal LCS referral by encouraging primary care appointments or risk assessment tool use.

Professionals' initial face‐to‐face contact within the LCS pathway was considered key, with rapport potentially facilitating remote follow‐up. Some experts preferred cessation discussions *before* LDCT scans, as receiving scan results at later stages could be stressful. They suggested providing varied low‐intensity supports for participants' consideration and ensuring continued follow‐up to maintain engagement across stages.

##### 3.3.2.2 Risk Assessment

To maximise participant reach, experts suggested incorporating cessation‐related assessments (e.g., readiness to quit) within LCS eligibility tools. One expert suggested emphasising key messages regarding combination pharmacotherapy and behavioural counselling rather than offering ‘too many’ personalised support options.

##### 3.3.2.3 Scan and Receipt of Results

Some experts explicitly identified the LDCT scan timepoint as well‐suited to smoking cessation‐related discussions. There were mixed views on discussions *after* receipt of results: some assumed that participants may be more amenable, while others suggested consistent messaging around cessation benefits to minimise providing any potential ‘licence to smoke’.

##### 3.3.2.4 Continuing in Screening Program (and ‘Beyond Screening’)

Rather than LCS being ‘one‐off’, experts discussed potential opportunities for continued support. They suggested initiating regular repeated contact to ‘check in’ and ensuring after‐hours service availability. However, some were concerned that actual repeat participation may be suboptimal.

#### 3.3.3 Cross‐Cutting Considerations

##### 3.3.3.1 Smoking Cessation Intervention Types

Experts discussed a range of potential interventions throughout. They frequently noted brief/very brief smoking cessation intervention as the minimum that should be offered to all participants, to ‘constantly remind’ participants throughout encounters and particularly when longer counselling was not feasible. LCS population‐tailored programs had strong support, including age group‐specific advice (e.g., ‘*after the age of 55, there is an exponential increased risk of illness…*’ [#15, program manager]). There was general recognition that online/phone/app‐based systems would be well‐received as complements to other supports, and that anonymised options could promote open disclosure. Motivational interviewing was sometimes suggested to encourage participant reflection, with the ‘full’ approach sometimes considered suitable during dedicated LCS appointments and shorter opportunities also noted to personalise cessation advice. Experts felt that contingency management interventions (i.e., regular financial/other incentives for verified non‐smoking) [42] could motivate service engagement or cessation attempts ‘*that otherwise wouldn't start*’ [#08, researcher], but expected greater controversy in LCS than in existing small‐scale implementation in other contexts.

##### 3.3.3.2 Service Delivery Modes

Existing differences between and within services were noted in interviews (e.g., infrastructure, local service availability), and experts encouraged assessing site‐specific needs to support consistent delivery in any given model. To maximise acceptability of cessation interventions, experts recommended local tailoring by leveraging service partnerships and co‐designing. Other strategies with strong expert support included scheduling convenient appointments via telehealth and allowing continuity of care with the same professionals where possible. Through potential models, there was very strong endorsement of opt‐out intervention offers to all LCS participants currently smoking, irrespective of readiness to quit [43]. Experts suggested routine framing within standard LCS protocols (e.g., ‘*how we're going to help you stop smoking…*’ [#12, advisor]), but acknowledged opting out should remain straightforward to avoid expectations of repeated cessation‐related discussions impeding re‐engagement.

##### 3.3.3.3 Communication and Messaging

The potential for targeting of recruitment communications (with LCS‐eligible consumer feedback to ensure appropriateness), and disseminate messaging through age‐appropriate channels (like social media and in entertainment venues) was suggested through interviews. Throughout LCS encounters, experts recommended informal rapport building, active listening, and a non‐judgemental tone. Experts also felt that a broader range of preventive health issues could be communicated during LCS‐related encounters. Further, aligned smoking‐related information was suggested to reach LCS‐ineligible individuals, to celebrate continued abstinence for those who had quit long ago and prevent smoking initiation for younger people.

##### 3.3.3.4 Other Program Requirements for Smoking Cessation, and Other Delivery Considerations

Experts frequently discussed smoking history data capture requirements within and outside of LCS to support smoking cessation and optimise workflow. They suggested that tools should: embed within existing systems, document intervention offers, and facilitate follow‐up discussion. Documenting priority population background in referrals was a specific strategy identified to support appropriate intervention delivery via external services. Experts strongly supported integration to avoid system ‘competition’, and highlighted effective communication and coordination within and across systems as essential. They recommended secure information sharing across providers and presenting services as integrated to participants regardless of the delivering organisation. Experts recommended regular data monitoring and reporting against performance targets, particularly in early LCS implementation, and evaluation and audit requirements to promote service accountability; LCS participant and community (qualitative) feedback was emphasised as an essential complement. Longer‐term quality improvement through piloting and trialling new strategies was also suggested.

##### 3.3.3.5 Workforce and Training Requirements

Almost all experts strongly supported professional training and upskilling, to promote contemporary, evidence‐based cessation intervention delivery. Experts suggested that training avoid a focus on individual blame by ensuring coverage of tobacco industry influence, health inequities, and the biological basis of nicotine dependence; they also recommended applied clinical anecdotes to enhance perceived professional relevance. Experts recognised cultural appropriateness training as important for staff, plus bilingual counsellors and cultural liaison officers to improve priority population support. Local smoking cessation champions were also suggested to ‘*take the lead*’ [#21, clinician] by facilitating peer support within services and promoting community LCS/smoking cessation efforts. However, experts cautioned against reliance on individuals, to reinforce smoking cessation as an ongoing system‐wide priority. They highlighted the importance of role clarity and securing senior service leadership buy‐in, along with recognition of the time required for cessation intervention.

## 4 Discussion

### 4.1 Main Findings

This study was conducted prior to the Australian Government's commitment to the National LCS Program, and unsurprisingly, strong support was expressed for LCS implementation and associated smoking cessation interventions among this expert sample. Similar to previous professional group reports [44, 45], experts anticipated LCS participants' motivation to quit would increase in an organised program. They also noted potential complements to existing initiatives for the LCS‐eligible population, and emphasised comprehensively continuing both individual smoking cessation and population‐level tobacco control.

### 4.2 Implementation Barriers and Facilitators

Consistent with previous studies [44, 46, 47], experts described stigma as a key barrier and suggested addressing it in LCS communications to minimise disengagement. Providing recommended verbal communication guidance to professionals (e.g., what to ‘do and not do’ [48]) was a specific expert‐identified strategy. Resources supporting provider‐participant discussion and avoiding inadvertent stigma exacerbation [49, 50] can minimise perceived judgement [51]. This should also inform cessation information developed for use in LCS program promotion and recruitment [52]. Reinforcing that smoking cessation is generally increased in LCS may also address perceived fatalism. Specific priority population‐targeted supports were also recommended, especially through co‐designed, strengths‐based approaches with potential to reach those supporting LCS participants, consistent with other tobacco control expert studies [53, 54].

### 4.3 Potential Delivery Models

Experts showed very little support for cessation intervention delivery via external service referral alone, and recognised that some degree of service coordination and delivery *within* LCS could maximise participants' cessation intervention (and particularly pharmacotherapy) access. International perspectives similarly caution that external service reliance could limit outcomes [55] or even exacerbate inequality [18]. These findings suggest consideration of planning and developing smoking cessation integration into Australian LCS.

### 4.4 Study Implications

#### 4.4.1 Informing Policy and Program Design

Strategies suggested to address barriers and support implementation included leveraging existing community health service connections, and LCS recruitment through smoking cessation services where respiratory health may be highly salient [44]. Some studies have found cessation service recruitment has successfully facilitated initial LCS engagement, particularly for priority population groups [56]. The professional community may be open to this strategy; an Australian trial is currently testing feasibility via telephone Quitline and may guide future implementation [57].

LCS implementation presents new opportunities, such as to assess a broader range of cessation‐related outcomes and to discuss smoking cessation at multiple occasions of the LCS screening and assessment pathway. Consistent messaging may also promote the importance of smoking cessation, with resources that counter negative expectations to avoid disengagement from people who smoke. These findings are particularly relevant to LCS workforce development and should inform the development of comprehensive staff training [58]. Professional education can also be extended to health providers not directly involved with LCS implementation. Experts emphasised the importance of regular, guideline‐consistent offers of combination pharmacotherapy and behavioural counselling services, rather than limiting intervention provision across the LCS pathway. Within LCS trials, higher quit rates are observed with more intensive interventions [21, 29, 59, 60].

#### 4.4.2 Implementation in Australia

Increasing evidence‐based cessation intervention access is one National Tobacco Strategy (2023–2030) objective, and to maximise support for those currently smoking, regardless of LCS eligibility, Cancer Council Australia's position statement emphasises embedding evidence‐based cessation interventions within both LCS *and* routine care in other health contexts [61]. Adequate clinical time for providing cessation interventions is a determinant widely recognised in LCS contexts [13, 62, 63, 64], and to overcome time pressures, experts also recommended dedicated cessation professionals within the program. Nurse navigators can increase cessation intervention delivery within LCS [65] and these roles may be feasible within designated centres or some other organised program contexts; for example, BreastScreen Australia screening services directly employ dedicated staff [66]. Australia will mostly have a decentralised delivery model, however, with LDCT scans accessed at private radiology services. Providers will deliver the Australian National LCS Program alongside a broader range of (non‐LCS) radiological services [23], which may limit potential opportunities to deliver cessation interventions at the time of the LDCT scan.

### 4.5 Strengths, Limitations, and Future Research

Building on previous pre‐implementation research [11, 12, 13, 67], this is the first Australian study to consider tobacco control experts' views. Although the potential age‐based eligibility described in interviews differs from the current National LCS Program criterion (50–70 years for all participants), experts generally only discussed this population broadly and placed greater emphasis on > 30 pack‐year smoking history defining LCS eligibility.

As one of the few international examples considering how best to provide support in this context, and the first such study prior to National Program funding, this research extends a limited evidence base through in‐depth qualitative exploration of implementation strategies and has direct relevance to LCS policy and program design. We document a range of expert‐identified implementation strategies to support intervention delivery during LCS; our mapping of key implementation determinant topics, across all CFIR domains, can enhance generalisability in other areas [68]. Experts' strong support of learning from existing overseas implementation experience suggests that future research considering and comparing expert perspectives across countries would be highly informative. Generating evidence in the Australian LCS context (e.g., on intervention effectiveness) and adapting potentially suitable cessation resources using co‐design approaches should now be prioritised. Early international trials showed strong LCS participant support for integrated smoking cessation intervention [69], and to date, broader studies with Australian LCS candidates/trial participants have considered broad perspectives on cessation intervention [67, 70, 71]. Yet, relatively little is known about how specific interventions would be received in this context and we recognise that LCS participants' views will not necessarily align with experts' [12]. However, our foundational evidence may be extended by seeking consumer perspectives on the acceptability of expert‐identified strategies and associated cessation‐related communications such as via mixed methods designs.

## 5 Conclusions

Australian tobacco control experts advocated for implementing routine offers of smoking cessation interventions in LCS, and potential strategies for consideration in program development, including overcoming key determinants in current service delivery. The findings emphasise the importance of building dedicated infrastructure for cessation interventions into routine delivery to realise these opportunities within the National LCS Program. Findings are highly relevant to the planning of international LCS programs and implementing optimal cessation intervention delivery within them. LCS has the potential to facilitate repeated smoking cessation intervention opportunities, including in ways that overcome existing system barriers, and to reinforce cessation initiatives in non‐LCS contexts. To maximise outcomes, cessation interventions delivered in the context of an Australian LCS program should be provided through LCS‐specific services rather than relying on external services alone.

## Author Contributions

**Nathan J. Harrison:** data curation, formal analysis, investigation, methodology, writing – original draft. **Diane Riddiford‐Harland:** conceptualisation, data curation, formal analysis, investigation, methodology, writing – review and editing. **Sarah York:** conceptualisation, data curation, formal analysis, investigation, methodology, writing – review and editing. **Henry M. Marshall:** conceptualisation, funding acquisition, methodology, writing – review and editing. **Joel J. Rhee:** conceptualisation, funding acquisition, methodology, supervision, writing – review and editing. **Emily Stone:** conceptualisation, funding acquisition, methodology, writing – review and editing. **Mei Ling Yap:** conceptualisation, funding acquisition, methodology, writing – review and editing. **Ashleigh R. Sharman:** conceptualisation, formal analysis, methodology, writing – review and editing. **Marianne Weber:** conceptualisation, methodology, writing – review and editing. **Susan A. McCullough:** conceptualisation, formal analysis, methodology, writing – review and editing. **Terri Byrne:** conceptualisation, formal analysis, methodology, writing – review and editing. **Christine L. Paul:** methodology, supervision, writing – review and editing. **Jacqueline A. Bowden:** methodology, supervision, writing – review and editing. **Billie Bonevski:** conceptualisation, methodology, supervision, writing – review and editing. **Nicole M. Rankin:** conceptualisation, formal analysis, funding acquisition, investigation, methodology, supervision, writing – review and editing. Nathan J. Harrison, Sarah York, and Nicole M. Rankin had direct access to the underlying data and verified reporting in the manuscript. All authors approved the final manuscript.

## Disclosure

Declaration of Generative AI and AI‐Assisted Technologies in the Writing Process: During the preparation of this work, the authors used GPT‐4o (via ChatGPT) in order to edit sections of the manuscript and improve readability. After using this tool, the authors reviewed and edited the content as needed and took full responsibility for the content of the publication.

## Ethics Statement

The study was approved by the Human Research Ethics Committee of The University of Sydney (2021/850), in line with the National Statement on Ethical Conduct in Human Research, ratified by University of Melbourne Human Ethics (2023‐25412‐40209‐5), with Cross‐Institutional Approval recognised by the Flinders University Human Research Ethics Committee (6343).

## Consent

Informed consent was documented in the online survey, including for audio recording of interviews. Consent was verbally affirmed at interviews, or implied by interview participation for those who did not complete the survey in advance.

## Conflicts of Interest

N.J.H. reports support for travel from Thoracic Oncology Group Australasia, payment to their institution from Lung Foundation Australia, and honoraria from Kom op tegen Kanker (Flemish Cancer Society, Belgium), outside of the submitted work. H.M.M. reports honoraria from Astra Zeneca, funding to their institution from the National Health and Medical Research Council and Medical Research Future Fund for lung cancer screening‐related research, that they are a National Lung Cancer Screening Program Expert Advisory Committee member with the Department of Health and Aged Care, and tobacco control committee member with the Thoracic Society of Australia and New Zealand, International Association for the Study of Lung Cancer, and Asian Pacific Society of Respirology. E.S. reports honoraria from AstraZeneca, Merck Sharp and Dohme, and The Limbic, support for attending meetings and/or travel from Astra Zeneca, and ad hoc advisory board payment from Bristol Myers Squibb. M.L.Y. reports funding to their institution from the National Health and Medical Research Council. M.W. reports honoraria from the Japanese Cancer Association, contract funding to their institution from Cancer Australia for lung cancer screening‐related work, and funding to their institution from the Medical Research Future Fund and National Institutes of Health outside of the submitted work.

## Supporting information


Data S1.

## Data Availability

The data generated in this study cannot be shared because of privacy or ethical restrictions. Public availability may compromise expert participants' confidentiality or reveal confidential information about their employers.
