# Supplementary material for: ‘Every Touch Point Is an Opportunity’: Tobacco Control Experts' Views on How to Implement Smoking Cessation Interventions Within an Australian Lung Cancer Screening Program
Source: Cancer Med. 2025 May 19;14(10):e70963. doi: 10.1002/cam4.70963 (PMC12086983; doi:10.1002/cam4.70963)
Supplement: Supplementary file 1 — Data S1. [file CAM4-14-e70963-s001.docx]

**Additional File 1**

Smoking Cessation in LDCT Lung Cancer Screening: Interview Schedule and Focus Group Guide

**Focus question: How can smoking cessation interventions be optimally embedded into an organised lung cancer screening program in Australia?**

Introduction script for interviewer / facilitator:

Hello, my name is [interviewer] from the University of [name],

**Interview:** Can I please speak with [participant name]?

Thank you for taking the time to talk with me today. We scheduled this time for an interview to discuss your thoughts about including smoking cessation within an organised lung cancer screening program. The interview will take about 45 minutes. Is now still a good time to talk?

[*If yes, then continue; if no, then reschedule*]

Hello, my name is [facilitator] from the University of [name],

**Focus group:** Welcome today everyone. Thank you so much for being part of this focus group to discuss thoughts about including smoking cessation within an organised lung cancer screening program. The session today will run for about 45 minutes. Is everyone happy to go ahead?

[*If yes, then continue; if no, then reschedule*]

**Interview and focus group:** I would like to make an audio recording, to be sure I accurately record what we talk about. Is this ok? I assure you, the recording will be kept strictly confidential.

*[If participant(s) agree, start recording – and remind participant about agreement to recording via consent documentation]*

Introduction to topic

Before we begin, just a brief context to today’s questions. As you are aware, our research group is investigating embedding smoking cessation within lung cancer screening - and what I am hoping to talk to you about today is how best to implement these smoking cessation interventions into an organised screening program.

As you may know, lung cancer screening focuses on people at high-risk rather than being population-based. The two largest international trials are the NLST (conducted in the US, reported in 2011) and NELSON (conducted in Europe and reported in 2020) used low dose computed tomography (or low does CT) to detect lung nodules. The eligibility criteria for these trials included age and smoking history, that is, typically people aged between 55-74 years, who are current smokers and have a pack-year history of 30 or more years. People who are former smokers with a similar history and have quit in the past 15 years are also eligible.

The two trials I mentioned reduced lung cancer mortality by up to 24% and demonstrated clinical effectiveness in diagnosing cancers at an early stage (known as stage shift). Lung cancer screening programs have been implemented across the US and South Korea and are being pilot tested in several countries. Many of these programs have offered smoking cessation interventions, but the evidence is not yet conclusive in terms of how best to embed smoking cessation with an organised lung cancer screening program.

So I would really like to explore what you (as a key stakeholder) think is required to successfully integrate smoking cessation into the screening pathway. We are interested in your thoughts, opinions and what you think might be feasible and acceptable to engage people at high-risk in smoking cessation as part of an organised lung cancer screening program.

Do you have any questions before we begin?

**Table S1. *Smoking cessation stakeholder qualitative study: interview schedule and focus group guide***

| ***Experience with smoking cessation/tobacco control*** | ***Additional information for the interviewer/facilitator to use:*** |
| --- | --- |
| 1. In what capacity have you worked in smoking cessation/tobacco control? Or, what is your role? 2. How many years’ experience have you had working in this area? 3. Where are you located to fulfil this role? E.g. Hospital/ university 4. What do you think works best for people attending smoking cessation programs at the moment? | *Prompt for q4. Do the current approaches result in optimal health outcomes for the community? I.e. people quitting or a reduction in smoking intensity (why/why not?)* |
| ***Smoking cessation within lung cancer screening*** |  |
| 1. What are your thoughts about embedding smoking cessation interventions within a lung screening program? 2. How do you think people who currently smoke would feel about being approached during a lung screening program?   *Prompts:* Who might refuse to participate? Should it be an opt-out approach? (*opt-out is where cessation advice is offered to all participants unless an individual indicates that they want don’t want to participate*)   1. What do you think would be the preferences of people who smoke for participating in a smoking cessation program at the time of screening? 2. How do you think we should tackle important groups or ‘priority populations’ including people from:  - Culturally and linguistically diverse backgrounds? - Aboriginal &Torres Strait Islander communities? - Rural and remote communities? - who may not want to stop smoking?   *Prompts:* Are there any particular sensitivities that need to be considered for such people?   1. What smoking cessation resources need to be co-designed with such communities? | *If the participant asks, here is some background information about evidence for cessation within an organised LCS program:*  A recent systematic review acknowledged that providing smoking cessation interventions within organised lung cancer screening programs offers an opportunity to reduce smoking-related morbidity and mortality for people ‘high-risk’. But the review noted that currently, the optimal strategy for delivering such interventions in a screening context is not clear (reference: Iaccarino et al. 2019)  The review suggested a two-pronged approach of combined counselling and pharmacotherapy to optimise cessation during screening but that more research with a focus on evaluating effectiveness and implementation is needed.  A second review, published in 2020 identified several promising approaches - also finding that multimodal interventions are likely to be more effective than single-modality interventions, although perhaps more costly. |
| ***Potential approaches: smoking cessation program within LCS*** | ***Additional information for the interviewer/facilitator to use:*** |
| As I mentioned earlier, eligibility in an organised lung screening program would target high-risk people aged 55-74 years (and Aboriginal and Torres Strait Islander peoples from 50-74 years).  I would like to discuss three (3) possible approaches or pathways about how to embed cessation into screening and seek your thoughts about these approaches*.*   - **1st** option: A cessation program completely embedded within LCS program where all cessation services and resources are coordinated and delivered within the program - **2nd** option: A cessation program wholly external to LCS and delivered by an agency like Quit NSW. - **3rd** option: A hybrid model of a cessation program where some cessation services or advice is embedded inside the screening program, but other aspects might require a referral to an outside agency (like Quit NSW).  1. How successful do you think these three (3) approaches might be?   *Prompts: I’d like to concentrate first on factors that could facilitate the inclusion of smoking cessation. What factors do you think might be barriers to including cessation interventions?*   1. Do you have any other suggestions or alternatives to these potential approaches?   *Prompt: how often do you think participants should be offered cessation advice?*  *Who should deliver cessation interventions/advice?*  *What kind of training and education is cessation is needed for staff in these options?*   1. How should cessation be offered for people who are returning for repeat-screening appointments? | Option 1: E.g. own cessation counsellors or trained nurses who are employed by program, participant follow up is within program  Option 2: E.g. referral out to primary care, Quitline and other existing resources in the community, pharmacy; LCS staff are primarily in a role as a *referrer*, responding based on participant’s needs and preferences  Option 3: E.g. LCS program staff are given specialised training in how to provide cessation advice, motivational interviewing, non-judgemental communication; primary care and other referrers into the program are also provided with a specialised training about LCS and cessation. |
| ***Policy and health system considerations*** | ***Prompts for this section*** |
| In the final part of the discussion, I’d like to get your thoughts on the broader health system and policy considerations regarding smoking cessation within the setting of lung cancer screening.   1. What would you say is the most important policy factors in optimally embedding smoking cessation into an organised lung cancer screening program? 2. What do you think would be the evidence base needed to support embedding cessation? 3. What would be the most significant barriers to the successful implementation of a smoking cessation program within lung screening? 4. What would be the most significant enablers to the successful implementation of a smoking cessation program within lung screening? 5. What are the most significant challenges around delivery of cessation services in the setting of lung cancer screening, based on your experiences? 6. How is lung cancer screening offered to people in smoking cessation programs (i.e. attracting people who might be eligible but unaware of LCS to consider enrolling in an organised program) 7. What is your perspective on vaping and e-cigarettes as smoking cessation interventions that might be part of an organized lung screening program? 8. To finish, is there anything else that you’d like to discuss or suggest on the topic? | *Do you think the scientific evidence in support of SC is enough to encourage policy change?*  *Findings from international studies? E.g. is longitudinal follow up data from major screening RCTs required?*  *I.e. including mortality outcomes and cost effectiveness?*  *What do you think about cost-effectiveness and what are the most important considerations?*  *Scalable and adaptable to different settings (e.g. in person vs. virtual resources for people who access mobile screening and then need follow up* |

I really appreciate you speaking with me today and sharing your views.

We are looking to get a broad scope of opinions.

Please may I ask if there any colleagues you can think of who might be interested in participating in this study that we could invite to participate? *(If yes, ask for contact details or send follow-up email to acquire this information*).

Once again, thank you for your time and, as mentioned in the Participant Information Statement, a gift voucher will be mailed to you in the next 6-8 weeks as a token of our appreciation.

**Additional File 2**

**Table S2.** Key topics and associated coding structure, with corresponding Consolidated Framework for Implementation Research (CFIR) domains as relevant.

| **High-level code** / Code | **What code represents?** (e.g., noted alignment with relevant CFIR domain/construct, other theory, or content areas) | **What ideas should be included in it?** (based on initial inductive coding from analysis team) |
| --- | --- | --- |
| **1. (IMPLEMENTATION) BARRIERS** | Multiple CFIR domains |  |
| 1.1. Issues with implementation (implementation IS the challenge) |  | - Every health care appointment with a smoker does not yet include smoking cessation advice  - Differences between jurisdictions |
| 1.2. Attitudes – clinicians | (CFIR **Individuals- Characteristics** subdomain – individual HPs are committed to fulfilling smoking cessation roles) | - Health professional perception that cessation intervention is offered too frequently (repeatedly)  - Health professionals don’t want to be seen as nagging |
| 1.3. System barriers to providing cessation intervention |  | - Health professionals have competing priorities  - Offering smoking cessation intervention is a choice; it requires the will and commitment  - Getting more time to offer cessation intervention needs more money  - Resource intensiveness  - Standard service hours will limit service access  - Offering smoking cessation intervention needs to be a billable service  - All Quitlines should be toll free  - Embedded cessation intervention has not been implemented successfully in other (Australian) contexts  - System needs to support health professionals doing their jobs  - Setting up a program takes time  - Additional steps in a new service (people they don’t know) |
| 1.4. Repeat quit messaging/ repetition of |  |  |
| 1.5. Insidious nature of tobacco industry | (including **Outer Setting- External Pressure** subdomain and Market Pressure construct – competing entities [counter] the implementation and/ or delivery of the innovation) | - Tobacco industry (still) has influence in health care |
| 1.6. Nicotine dependence - quitting smoking is hard |  | - Need to acknowledge addiction - smoking is not an (individual) lifestyle choice  - Other substance issues complicate (nicotine) dependence  - Managing nicotine dependence should be a primary focus of cessation intervention |
| 1.7. Potential for LCS to reinforce continued smoking |  |  |
| 1.8. Access barriers |  | - Barriers to treatment reduce likelihood of quitting  - Mobile and internet limitations in rural and remote areas  - Mobile screening in rural and remote areas |
| **2. COMMUNICATION / MESSAGING** | CFIR **Inner Setting** domain- Communications - there are high quality formal and informal information sharing practices within and across Inner Setting boundaries (e.g., structural, professional) |  |
| 2.1. Using existing smoking cessation programs to promote LCS |  | - Screening extends the reach of existing tobacco treatment services  - Screening as an (inherent) part of smoking cessation  - Group program participants are motivated (receptive to offer of new programs)  - Referral to LCS via smoking cessation services alone is not sufficient |
| 2.2. Quitline use – to communicate/ recruit for LCS |  | - Quitline as entry point and can also facilitate referral for LCS  - Potential to also refer to LCS through 13 11 20 [Cancer Council Information and Support service] |
| 2.3. Engagement strategy through health communication/ messaging |  | - LCS invitation needs broad communications (not just via smoking cessation services)  - Communications need to target smokers rather than focus on general population  - Need to maximise cost-effectiveness of communications  - The limitations of LCS need to be communicated clearly and honestly  - Framing success of other screening programs in messaging  - Need to increase awareness that LCS can improve lung cancer outcomes  - Communications first need to focus on information and raising awareness  - Make clear that screening not affected by smoking cessation choices  - Consistent and continued messaging required  - Use success to spread the word about cessation |
| 2.4. Awareness of screening and treatment options |  | - Communications (can) prompt referral |
| 2.5. Proactive approaches to maximise reach of program | Where the proactive approach is not otherwise specified/aligned with another, more specific code |  |
| 2.6. Other health promotion strategies |  |  |
| **3. (IMPLEMENTATION) ENABLERS / FACILITATORS** | Multiple CFIR domains |  |
| 3.1. Implementation of existing resources – resources are not a barrier |  | - Lots of tobacco control resources have been developed already  - Everyone knows that smoking is bad for health  - LCS is just another program to offer smoking cessation intervention  - People who smoke are already exposed to broader tobacco control policy responses  - Need to look at how to use existing resources: working well with what you already have, not re-inventing resources  - Frustration/fatigue with continuing to develop unneeded resources  - Written materials alone are not sufficient |
| 3.2. Online/app-based interventions |  | - Cheap/cost effective  - Increases access to intervention  - Evidence for online intervention is building |
| 3.3. Suite of options/Lots of treatment types |  |  |
| 3.4. Offering cessation intervention in LCS increases re-screening |  |  |
| 3.5. Local champions make it happen | (including CFIR **Individuals- Roles** subdomain and Implementation Leads construct - individuals who lead efforts to implement the innovation) | - Getting embedded care requires champion staff |
| 3.6. Patients’ existing engagement with healthcare system |  | - Older people are more likely to use health services  - Not everyone has a (regular) GP  - Alcohol and other drug services (that take referrals) are ‘permanent’  - LCS population is not well-supported in general  - Inclusion of primary care |
| 3.7. Education and awareness as a precursor to behaviour change |  |  |
| 3.8. LCS/smoking cessation in a LCS context can reinforce broader tobacco control responses | (including **Outer Setting- External Pressure** subdomain and Societal Pressure construct - mass media campaigns, advocacy groups, or social movements or protests drive implementation and/or delivery of smoking cessation) | - Anti-tobacco mass media promotes use of cessation services  - Bombarding with health messages is not effective |
| 3.9. System integration - communication |  | - LCS/smoking cessation implementation should not be standalone |
| **4. EVIDENCE BASE FOR LCS** | CFIR **Innovation** domain-Innovation Evidence Base construct – LCS has robust evidence supporting its effectiveness |  |
| 4.1. Perception that early evidence did not support benefits of LCS (out of date) |  |  |
| 4.2. New/ current LCS evidence update – good effectiveness |  |  |
| 4.3. Evidence for smoking cessation within LCS |  | - Need to generate local evidence on LCS use of smoking cessation intervention |
| **5. EXAMPLES OF SUCCESS** | CFIR **Innovation** domain-Innovation Evidence Base – LCS has robust evidence supporting its effectiveness |  |
| 5.1. Optimal example/ model |  | - LCS as part of a lung cancer optimal care pathway (more broadly) |
| 5.2. Learning lessons from experience |  | - Learning from other screening programs |
| 5.3. Models working well overseas |  | - Australian systems are similar to those overseas  - Funding services overseas has multiple layers too  - Local levels of government implement services overseas too  - Some regions overseas do better than others |
| 5.4. Australia has demonstrated (some) tobacco control success | (CFIR **Outer Setting** domain-Local Conditions - economic, environmental, political, and/or technological conditions related to smoking cessation [do or do not] enable the Outer Setting to support implementation and/or delivery of the innovation) | - There is still progress to make in tobacco control  - Current quit rates are not high |
| **6. OTHER LCS PROGRAM REQUIREMENTS FOR SMOKING CESSATION** | Multiple CFIR domains |  |
| 6.1. Data capture in LCS program | (CFIR **Inner Setting** domain- Information Technology Infrastructure - Technological systems for tele-communication, electronic documentation, and data storage, management, reporting, and analysis support and functional performance of the Inner Setting). This code should relate to *tracking* of smoking cessation services - not about people attending their next LCS appointment | - IT & systems  - Tracking  - Centralised data collection  - A centralised database is required for the LCS program to coordinate  - Electronic medical systems differ between jurisdictions |
| 6.2. Data requirements outside of LCS program | (CFIR **Inner Setting** domain- Information Technology Infrastructure) | - Embedded (electronic) records will facilitate follow-up  - Referral should be electronic  - GP practice software to record smoking history and prompt for LCS referral  - Data needs to be within GP practice software in order to be used |
| 6.3. Reporting requirements | (CFIR **Implementation Process** domain-Reflecting & Evaluating - Collect and discuss quantitative and qualitative information about the success of implementation and/or the innovation) | - Mandatory program KPIs need to be set and reported on  - Responsible reporting  - Reporting at subgroup levels to explore priority population group trends  - Know what is being done and report back to organisation |
| 6.4. Accountability | (CFIR **Implementation Process** domain-Reflecting & Evaluating [Implementation]- Collect and discuss quantitative and qualitative information about the success of implementation) | - Program quality needs to be a priority |
| 6.5. Evaluation/audit is required | (CFIR **Implementation Process** domain-Reflecting & Evaluating [Implementation]) | - Evaluation methods/examples |
| 6.6. Other policy | Code for policies beyond the level of individual health services or networks – e.g., national program, state, professional organisations | - National approach |
| **7. *PARTICIPANT* BELIEFS** | CFIR **Individuals- Characteristics** subdomain – the characteristics of individuals |  |
| 7.1. Self as a smoker |  | - Those who don’t identify as ‘smokers’ are at risk and need to be included |
| 7.2. Participant awareness |  |  |
| 7.3. Perception of benefits |  | - Misconception re. mortality benefits  - Benefits compared to other cancer types  - LCS is unlike other screening programs [breast, bowel, cervical cancers] |
| 7.4. A requirement of LCS is quitting |  | - Hope that quitting is not a requirement of screening |
| 7.5. LCS is scary |  | - Talking about cancer can turn some people away  - Preference for ‘lung health check’ term [as used in England] |
| **8. PARTICIPANT EXPECTATIONS - SCREENING AS PART OF CESSATION EFFORTS** | CFIR **Individuals**-Characteristics subdomain – the characteristics of individuals |  |
| 8.1. ‘Haunting’ history/legacy - worry about smoking-related illness persists after quitting |  |  |
| 8.2. Smokers experience negative emotions around quitting |  |  |
| 8.3. Motivation to quit | (Motivation construct – individual LCS participants are committed to engaging in smoking cessation) | - Health concerns will motivate quitting  - Building motivation to quit in cessation intervention  - Some participants will be resistant to smoking cessation |
| 8.4. People who smoke expect to be asked about smoking cessation |  | - Not asking about smoking suggests it is not important |
| 8.5. People who smoke expect cessation support from health care professionals |  | - Offering cessation intervention demonstrates patient care  - Cessation intervention needs to be delivered in a supportive way (open, empathetic, compassionate, etc.)  - Offering cessation intervention needs trust/familiarity [i.e., and unfamiliar environments will put participants off ever engaging] |
| **9. *PARTICIPANT* MOTIVATION** | CFIR **Individuals- Characteristics** subdomain and Motivation construct – individual participants are committed to engaging with smoking cessation |  |
| 9.1. Participant motivation to *screen* |  | - Willingness to participate in LCS is the first step – precursor to smoking cessation  - Motivated by LCS leading to better LC outcomes (e.g., through early detection)  - Different groups within groups |
| 9.2. Aware “early adopters of LCS” will be particularly ready to screen |  | - Selection bias – LCS participants will be more motivated to quit  - Smokers motivated to use cessation services will be honest about smoking  - “Early adopters” [of LCS] will be those who have already quit smoking |
| 9.3. Motivation *for smoking cessation* |  | - Most smokers want to quit already  - It’s fine if smokers are not yet ready to quit [i.e., they might be ready further down the pathway]  - “I like smoking” [i.e., a barrier for a person who smokes may be that smoking is considered pleasurable, or that they are not (yet) interested in quitting]  - Smoke-free environments promote use of cessation services  - Cost of smoking - quitting has huge financial gains  -- Price increases promote use of cessation services |
| **10. PATHWAY STAGE - ‘BEYOND SCREENING’: REPEAT AND FOLLOW-UP** | Considerations for this timepoint of the LCS screening and assessment pathway |  |
| 10.1. Continued and repeated engagement beyond screening |  | - Lung cancer treatment has opportunities to repeatedly include smoking cessation advice  - Smoking cessation intervention is often missing ‘help’ [i.e., the help step of the 3-step brief advice model]/follow-up  - Quitline can offer repeat follow-up to engage smokers  - LCS intervals are big |
| **11. PATHWAY STAGE - REFERRAL** | Considerations for this timepoint of the LCS screening and assessment pathway |  |
| 11.1. Key step is referral/initial approach |  | - Start with a focus on reducing not quitting  - Health care settings as an entry point |
| 11.2. Two directional pathway – referral between services (from LCS to smoking cessation AND smoking cessation to LCS) |  | - Potential for duplicate referral  - Feedback loop: external services provide feedback on contact to LCS  - Two-way communication |
| 11.3. External referral (to external smoking cessation services) |  | - Onus is on participants to use external services that follow-up on embedded LCS cessation intervention  - Not *just* referral: follow-up!  - External referral makes use of existing resources and processes  - External referral enhances 1-on-1 health professional interaction  - Need to supplement services with external referral  - Health professionals see referral as a tick box exercise  - Active referral  - Importance of warm hand off |
| **12. PATHWAY STAGE - SMOKING CESSATION ONCE PEOPLE ARE IN THE DOOR** | Considerations for this timepoint of the LCS screening and assessment pathway |  |
| 12.1. Approach for initial contact/appointment |  | - More detailed assessments (including for any past-month use, dependence)  - Connecting smokers with treatment is the important component  - Arrange connection to intervention and pharmacotherapy at the time of appointment |
| 12.2. Switching from LCS to cessation protocol |  | - Continuity/confined?  - Repeated engagement  - Communication  - LCS is a one-off encounter for smokers with a clear result (or no LC diagnosis) – follow through |
| 12.3. Getting people in the door |  | - Once smokers attend LCS, offering cessation is easy  - Tobacco control responses will not be effective if they don’t reach the target group |
| **13. PRIORITY POPULATIONS AND COMMUNITIES** | CFIR **Individuals**- Characteristics subdomain- Need – participants from priority population groups are disproportionately impacted by smoking harms, which will be addressed by implementation and/or delivery of LCS.  Code for considerations highlighted as specific to priority population groups, including but not limited to: people with culturally and linguistically diverse backgrounds, Aboriginal and Torres Strait Islander people, people living in regional/rural/remote areas |  |
| 13.1. Resourcing informational materials and contacts |  | - Need for tailored resources to show benefits of quitting  - Consumer input is important through the whole pathway (not just resource development) |
| 13.2. Unique risk factors for priority groups |  | - Different types of tobacco use (or other smoked products)  -- Need to pay more attention to use of non-combustible tobacco  - Sometimes those at highest risk are those most disengaged |
| 13.3. Needs to be inclusive of priority populations |  | - Services to provide culturally appropriate/safe care  - Self determination  - Specific subgroup needs |
| 13.4. Language is an enabler |  | - Funded translation is needed to meet local needs |
| 13.5. Historical legacy and barriers need to be addressed first |  | - Trust in health systems |
| 13.6. Stereotypes about priority populations | e.g., mental health, AOD use | - May also include perception that some people don't want to quit |
| **14. SERVICE DELIVERY MODES (FOR SMOKING CESSATION) IN LCS** | CFIR **Innovation- Innovation Adaptability** domain – smoking cessation interventions can be modified, tailored, or refined to fit local context or needs |  |
| 14.1. Opt in vs opt out |  | - An opt-out mechanism needs to be a natural component of LCS  - Smoking cessation cannot be forced  - Consent needed to offer cessation intervention  - Intervention ends after declined offer in opt-in |
| 14.2. Hybrid model makes operational sense |  | - Preference for hybrid system  - Referral within hybrid model makes operational sense  - A hybrid model maximises cost-effectiveness  - Setting up new services to do behavioural intervention would be very expensive |
| 14.3. Having the model work across different services | (CFIR **Inner Setting** domain) | - Consistency is needed across the multiple services delivering cessation intervention |
| 14.4. Site specific needs | (CFIR **Inner Setting** domain) | - Access to local (smoking cessation) services differs based on location  - Many local services are transient and then go  - Programs need both consistency and local adaptation  - Preference for local services (over Quitline) |
| 14.5. Quitline use – to deliver smoking cessation intervention |  | - Quitlines are specifically focused/dedicated on smoking cessation  - There is a level of consistency (in smoking cessation intervention) among state-based Quitlines  - The offerings of state-based Quitlines differ  - Quitlines are evidence-based  - Quitline is effective in rural and remote areas  - Perception that Quitline (just) tells you not to smoke  - Quitline offers low-intensity interventions  - Quitline has the capacity to take on extra referral from LCS (uptake is low) |
| **15. SMOKING CESSATION INTERVENTION TYPES** | CFIR **Innovation** domain- **Innovation Adaptability** construct – smoking cessation interventions can be modified, tailored, or refined to fit local context or needs.  Include in this code general principles/evidence of smoking cessation |  |
| 15.1. Every health care appointment with a smoker should include smoking cessation advice |  | - Smoking cessation not just part of LCS, part of wider health care system  - Inclusive supports |
| 15.2. Brief intervention models |  | - ‘3/5 As’ models for smoking cessation [‘3 As’ = 3-step brief advice model of smoking cessation intervention: ask, advise, help; similarly, ‘5 As’ = 5-step model: ask, advise, assess, assist, and arrange]  - Brief intervention should be the minimum level of cessation intervention offered  - Casual conversation leading to referral  - Risk of doing too much |
| 15.3. Normalising - smoking cessation intervention needs to be a natural component of LCS |  | - Smoking cessation embedded into LCS is a good idea  - Cessation and screening should go together  - Smoking cessation has been embedded into other activities  - Smoking cessation as an (inherent) part of screening  - Receiving intervention increases quit rates  - New smoking cessation resources required specifically for the LCS setting  - Ethical responsibility: not an option |
| 15.4. Tailored programs - Not one size fits all |  | - Most smokers quit without services  - Some will try to quit cold turkey  - Some will try to quit with other methods altogether  - Need to not discourage smokers from trying to quit with other methods altogether  - Many smokers do not want ongoing sessions with a counsellor  - Attrition from ongoing programs  - Preference (or reluctance) for online intervention over telephone-based or face-to-face  - Some have no strong preferences, and want help to quit regardless of form  - Gendered patterns in smoking behaviour  - Resources need to be (very) specific to location |
| 15.5. Progression of intervention - stepped approach |  | - Progressing through/repeating recommended interventions by intensity to improve self-efficacy  - Varying levels/stages of quitting (and readiness) will need different options  - Behavioural interventions need to cover stress management/replacement  - Importance of access to pharmacotherapy  -- Pharmacotherapy is more effective than online intervention  - Carbon monoxide detectors to track progress of cessation / give feedback back to smokers / facilitate referral  - Comprehensive/combined approaches required to achieve best outcomes  - Need to make as many resources as possible available  - In LCS, more intensive interventions are required |
| 15.6. Client centred model |  | - Need to consider networks of family and friends  - Cessation advice needs to consider social aspects of smoking and relationships |
| 15.7. Motivational interviewing |  | - Open-ended questions |
| 15.8. Solution-focused approach |  |  |
| **16. STIGMA (AND RELATED ATTITUDES)** | Multiple CFIR domains  Definitions from Rose et al.^(1)^*:* “According to Goffman's Stigma Theory, stigma is based on an attribute, characteristic or behaviour that devalues individuals or groups who are consequently seen as socially undesirable. Stigma can be perceived (i.e., the anticipation or fear of discrimination) or felt (i.e., discrimination from others), which can be internalised as feelings of shame, blame and guilt. In relation to lung cancer, the perceived controllability of disease onset due to known links with smoking can perpetuate stigmatising attitudes.” |  |
| 16.1. LCS participant level - stigma | e.g., internalised, self-stigma or self-blame. Individual level of Health Stigma and Discrimination Framework^(2)^ (knowledge, attitudes, etc.) | - Smoking-related stigma stops smokers from using services  - Smoking-related stigma changes smokers’ interactions with health professionals |
| 16.2. Other individual level - stigma | e.g., health professionals, other users of the service or group programs. Interpersonal level of Health Stigma and Discrimination Framework | - Smoking-related stigma is a health professional barrier to LCS  - LCS programs need to address smoking-related stigma |
| 16.3. Service level - stigma | e.g., institutions, LCS setting as a health professional workplace. Organisation level of Health Stigma and Discrimination Framework | - Patient resources reinforce stigma |
| 16.4. Community level - stigma | (including **Outer Setting- External Pressure** subdomain and Societal Pressure construct).  e.g., cultural values, norms, attitudes. Community level of Health Stigma and Discrimination Framework | - Advertising (commercial)/ anti-tobacco mass media reinforces stigma  - Lung cancer receives less attention despite worse outcomes - sympathy vs stigma for different cancer types  - Outdated thinking on smoking (e.g., personal flaw) |
| 16.5. Nihilism needs to be countered | LCS participant-perceived nihilism (specifically) unless better aligned with other individual, service, or community levels of stigma  *Nihilism*, from Chambers et al.^(3)^: “…in the context of lung cancer therapeutic nihilism is defined as the view that medical treatments for this illness are of no value.” |  |
| 16.6. Fatalism needs to be countered | LCS participant-perceived fatalism (specifically) unless better aligned with other individual, service, or community levels of stigma  *Fatalism,* from Portnoy et al.^(4)^: ” “…outcome-specific fatalism has often been operationalized as the belief about one’s efficacy to avoid that outcome…”, i.e. “lung cancer seemingly being caused by everything”. |  |
| **17. WORKFORCE REQUIREMENTS / TRAINING** | CFIR **Inner Setting** domain – the setting in which LCS is implemented (e.g., program site, associated smoking cessation service) |  |
| 17.1. Specialised training in addiction/smoking cessation and expertise is important for health professionals |  | - Clinicians educating each other  - Current referral is not optimised (requiring training)  - Need upskilling in primary care  - Detailed cessation advice can be provided to LCS participants, too |
| 17.2. Expectation of offering smoking cessation advice every time | (Culture construct - there are shared values, beliefs, and norms in offering smoking cessation) |  |
| 17.3. Expectation of what health care professional should deliver | (Culture construct) | - Need to include within clinical guidelines |
| 17.4. Roles and responsibilities - implementation requires role clarity |  | - Smoking cessation requires dedicated personnel (e.g., tobacco treatment specialists)  - Smoking easily skipped over because it’s not the presenting issue  - Involvement of specific professional groups |
| 17.5. Health professional motivation | (CFIR **Individuals- Characteristics** subdomain – individual HPs are committed to fulfilling smoking cessation roles) | - LCS staff will be particularly interested in smoking cessation / Other (non-LCS) staff will be particularly interested in smoking cessation  - LCS staff want to see outcomes change (poor survival)  - Health professionals are concerned about smoking  - Program success enhances health professional motivation |
| 17.6. Lived experience/engagement |  | - Need to include ‘smokers'’/real-world perspectives from the coalface  - Possible lack of *own* experience |
| 17.7. Buy in needs to come from all sides | (including CFIR **Individuals- Roles** subdomain and High-level leaders construct - individuals with a high level of authority, including key decision-makers, executive leaders, or directors) | - Implementation requires ownership  - Implementation requires commitment from the top  - Implementation requires committed funding |
| 17.8. LCS *awareness* is low among (tobacco control) professionals |  | - Training on LCS is needed |

***Note.*** High-level codes are in alphabetical order. The coding manual used in analysis also included exemplar quotations from the raw data, to illustrate codes; these are not included here to avoid excessive reproduction of the initial subset of transcripts used to develop the manual. During the codebook development, lower-level codes with shaded cells were assumed to align with the guidance listed for the higher-level code (e.g., in CFIR domain/construct, other theory, or content area alignment) unless otherwise specified. Content in [square brackets] is intended as a brief explanation of the codes developed or discussed within the analysis team. LCS = lung cancer screening; CFIR = Consolidated Framework for Implementation Research.

**References cited within supplementary files:**

^(1)^ Rose S, Boyes A, Kelly B, Cox M, Palazzi K, Paul C. Lung cancer stigma is a predictor for psychological distress: A longitudinal study. Psycho-Oncology 2021;30:1137–44. https://doi.org/10.1002/pon.5665.

^(2)^ Stangl AL. The Health Stigma and Discrimination Framework: a global, crosscutting framework to inform research, intervention development, and policy on health-related stigmas 2019:13.

^(3)^ Chambers SK, Dunn J, Occhipinti S, Hughes S, Baade P, Sinclair S, et al. A systematic review of the impact of stigma and nihilism on lung cancer outcomes. BMC Cancer 2012;12:184. https://doi.org/10.1186/1471-2407-12-184.

^(4)^ Portnoy DB, Leach CR, Kaufman AR, Moser RP, Alfano CM. Reduced Fatalism and Increased Prevention Behavior After Two High-Profile Lung Cancer Events 2014.
